# Breath biopsy for early detection and precision medicine in cancer

**DOI:** 10.3332/ecancer.2018.ed84

**Published:** 2018-07-19

**Authors:** Marc van der Schee, Hazel Pinheiro, Edoardo Gaude

**Affiliations:** Owlstone Medical Ltd, 183 Science Park, Cambridge, CB4 0GJ, UK

**Keywords:** biomarkers, breath, breathomics, precision medicine, personalised medicine, metabolism, lung cancer, colorectal cancer, oncogenesis, Warburg effect, early detection, metabolomics

## Abstract

Breath biopsy enables the non-invasive collection and analysis of volatile organic compounds (VOCs) in exhaled breath, providing valuable information about disease processes occurring in the body. Metabolic changes occur in cancer cells at the earliest stages of disease. We discuss progress in the use of breath biopsy for discovery of breath-based biomarkers for early detection of cancer, and potential applications for breath biopsy in enabling precision medicine in cancer.

## Introduction

Every breath contains a wealth of valuable information about the health of an individual, in the form of volatile organic compounds, or VOCs. Whereas genomic analysis provides information about disease predisposition, in reality disease is the result of multiple interacting genetic and environmental factors. The body’s metabolome is affected by all of these factors, and as such provides real time information which is closely related to actual disease activity.

Capturing VOCs in breath via a simple ‘breath biopsy’ provides a completely non-invasive means to analyse volatile metabolites produced by metabolic processes occurring throughout the body. Over 1,000 VOCs in breath have already been identified [1], representing a source of promising biomarkers for a wide range of diseases, including cancer.

With applications in early detection and precision medicine, breath biopsy has the potential to address two fundamental challenges in oncology today: firstly, early diagnosis of cancer patients, and secondly, precision medicine – ensuring patients receive the treatment which is most effective for them.

## Breath biomarkers for early detection of cancer

Cancer cells undergo profound changes in their metabolism in order to support high energetic demands of uncontrolled proliferation [2]. Several oncogenic mutations have been shown to affect the metabolism of cancer cells by converging to common metabolic pathways linked to cell cycle and anabolic growth [3–5]. The Warburg effect is among the well-established cancer metabolic hallmarks [6] and entails the activation of aerobic glycolysis as the main pathway for biosynthetic purposes, as opposed to normal cells that exploit mitochondrial metabolism for their energetic needs [7, 8].

These changes in cellular metabolism favour survival in an oxygen deprived environment and result in altered metabolic intermediates that function as the building blocks for new cells, both enabling the growth of rapidly dividing cancer cells, and also altering the profile of VOCs in breath [9]. As these processes are fundamental to cancer cell survival, such altered cellular metabolism occurs as one of the earliest stages of tumourigenesis [10–12], hence VOCs are excellent candidate biomarkers for early detection of cancer.

Reproducible breath collection in a clinical setting has been historically challenging, for example due to the use of polymer storage bags which result in low sensitivity for VOC detection, loss of VOCs through the bag, contamination with external VOCs, variable collection volumes, and carry-over on re-used materials [13], however a breath collection device developed in collaboration with over 100 breath diagnostics researchers [14] is now enabling the world’s largest breath-based clinical trial evaluating breath biopsy for early detection of lung cancer. The UK NHS funded LuCID trial (ClinicalTrials.gov identifier NCT02612532) [15] will recruit up to 4,000 patients, referred with clinical suspicion of lung cancer, and aims to identify a VOC biomarker signature in breath that can distinguish the presence or absence of lung cancer. It is hoped that a breath test would augment existing lung cancer screening techniques. Low-dose computed tomography (LDCT) suffers from a high rate of false positive results [16]. A breath biopsy test as a rule-in test would reduce unnecessary CT scans and invasive follow up procedures, reducing screening costs and increasing health benefits.

Breath biopsy is also being deployed in large scale clinical studies to identify biomarkers for the early detection of colorectal cancer (InTERCEPT project [17], involving up to 1,000 patients in collaboration with Warwick University and the University Hospital Coventry and Warwickshire NHS Trust) and multiple cancer types (PAN Cancer Study [18], in collaboration with Cancer Research UK).

## Precision medicine applications for breath biopsy

Since the VOC profile closely reflects metabolic processes, VOC biomarkers are well positioned to provide insight into the biological variability underlying disease processes. The potential for breath biomarkers in precision medicine applications has been mostly explored in the area of respiratory diseases such as asthma and COPD [19], and evidence suggesting VOCs in breath can differentiate between different subtypes of cancer [20] points towards the utility of breath biomarkers for precision medicine in cancer.

Metabolic pathways themselves are targets for the development of novel cancer therapies [21, 22], raising the possibility that breath biomarkers reflecting the target metabolic pathway may in future be used to stratify patients, enabling therapies to be directed to the patients most likely to benefit. Monitoring changes in the breath VOC profile before and during treatment could provide confirmation of therapy efficacy by tracking changes in disease-related VOC biomarkers, enabling treatment to be avoided or discontinued quickly if a lack of response is identified. Measuring specific drug-related volatile metabolites in breath may also provide valuable information about how a drug is being metabolised by the patient, which could be related to therapeutic response.

Continued mutations in cancer cells result in considerable heterogeneity in the tumour. Therapy failure is often associated with clonal expansion of a specific subset of tumour cells. The associated metabolic reprogramming [21, 23, 24], suggests that metabolic biomarkers could provide predictive information about therapy failure. Indeed, several lines of evidence indicate that metabolic flexibility is an important determinant of resistance to cancer therapies [25]. There is evidence from metabolomics analysis of tissue in pancreatic ductal adenocarcinoma patients that metabolic biomarkers may be predictive of resistance to the chemotherapy gemcitabine [26]. Metabolic changes may also be associated with drug-induced toxicity [27, 28], suggesting that VOC biomarkers in breath could be explored as potential predictors of toxicity.

Research is beginning to uncover the impact of the microbiome on oncogenesis and the efficacy of cancer therapies [29]. Recently, intratumour bacteria have been reported to metabolise a chemotherapeutic drug, leading to resistance [30]. The microbiome contributes to the body’s metabolome, and hence the profile of VOCs in breath can be analysed as a non-invasive means to discover biomarkers that reflect the activity or involvement of the microbiome in cancer.

## Conclusions

Breath biopsy provides a non-invasive way to capture and analyse volatile metabolites in breath, and gain a rapid insight into the metabolic changes associated with cancer from the earliest stages of disease. Large scale clinical trials are underway to evaluate breath biopsy for the early detection of multiple different tumour types. Building on the technological advances in breath collection and analysis that have been established, we can now begin to explore how breath biomarkers could help guide treatment decisions in a personalised medicine approach, for example by predicting drug resistance, toxicity and therapeutic response. Breath biopsy tests have the potential to revolutionise healthcare, enabling patients to be monitored over time without incurring significant costs or radiation exposure. The non-invasive nature of a breath test will ensure acceptability for patients, increasing uptake for screening programmes.
